# Cost of Medical Care of Patients with Advanced Serious Illness in Singapore (COMPASS): prospective cohort study protocol

**DOI:** 10.1186/s12885-018-4356-z

**Published:** 2018-04-23

**Authors:** Irene Teo, Ratna Singh, Chetna Malhotra, Semra Ozdemir, Rebecca A. Dent, Nesaretnam Barr Kumarakulasinghe, Wee Lee Yeo, Yin Bun Cheung, Rahul Malhotra, Ravindran Kanesvaran, Alethea Chung Pheng Yee, Noreen Chan, Huei Yaw Wu, Soh Mun Chin, Hum Yin Mei Allyn, Grace Meijuan Yang, Patricia Soek Hui Neo, Nivedita V. Nadkarni, Richard Harding, Eric A. Finkelstein

**Affiliations:** 1 Lien Centre for Palliative Care, Duke-NUS Medical School, Singapore, Singapore; 2 Program in Health Services Systems Research, Duke-NUS Medical School, Singapore, Singapore; 3 National Cancer Centre, Singapore, Singapore; 4 National University Cancer Institute, National University Hospital, Singapore, Singapore; 5 Tan Tock Seng Hospital, Singapore, Singapore; 6 Centre for Quantitative Medicine, Duke-NUS Medical School, Singapore, Singapore; 7 Dover Park Hospice, Singapore, Singapore; 8 King’s College London, Cicely Saunders Institute, London, UK

**Keywords:** Advanced cancer, End of life, Palliative care, Healthcare utilization, Singapore

## Abstract

**Background:**

Advanced cancer significantly impacts quality of life of patients and families as they cope with symptom burden, treatment decision-making, uncertainty and costs of treatment. In Singapore, information about the experiences of advanced cancer patients and families and the financial cost they incur for end-of-life care is lacking. Understanding of this information is needed to inform practice and policy to ensure continuity and affordability of care at the end of life. The primary objectives of the Cost of Medical Care of Patients with Advanced Serious Illness in Singapore (COMPASS) cohort study are to describe changes in quality of life and to quantify healthcare utilization and costs of patients with advanced cancer at the end of life. Secondary objectives are to investigate patient and caregiver preferences for diagnostic and prognostic information, preferences for end-of-life care, caregiver burden and perceived quality of care and to explore how these change as illness progresses and finally to measure bereavement adjustment. The purpose of this paper is to present the COMPASS protocol in order to promote scientific transparency.

**Methods:**

This cohort study recruits advanced cancer patients (*n* = 600) from outpatient medical oncology clinics at two public tertiary healthcare institutions in Singapore. Patients and their primary informal caregiver are surveyed every 3 months until patients’ death; caregivers are followed until 6 months post patient death. Patient medical and billing records are obtained and merged with patient survey data. The treating medical oncologists of participating patients are surveyed to obtain their beliefs regarding care delivery for the patient.

**Discussion:**

The study will allow combination of self-report, medical, and cost data from various sources to present a comprehensive picture of the end-of-life experience of advanced cancer patients in a unique Asian setting. This study is responsive to Singapore’s National Strategy for Palliative Care which aims to identify opportunities to meet the growing need for high quality care for Singapore’s aging population. Results will also be of interest to policy makers and researchers beyond Singapore who are interested to understand and improve the end-of-life experience of cancer patients.

**Trial registration:**

NCT02850640 (Prospectively registered on June 9, 2016).

## Background

Cancer is the primary cause of death in Singapore, accounting for approximately 30% of all deaths [1]. This proportion is expected to increase as the number of older persons ≥65 years of age is predicted to expand from 12.4% of the population in 2016 to 20% by 2030 [2]. Dealing with advanced cancer can be difficult for patients and families as they cope with symptom burden, treatment decision-making, loss of economic productivity, and financial costs of treatment [3–7]. This is especially true in Singapore if a patient were to deplete their Medisave savings (the national medical savings scheme) and given the limited coverage under Medishield Life (a national basic healthcare insurance scheme). Despite high costs of treatment, it has been reported that many patients with advanced cancer in Singapore often do not have their symptoms adequately managed, do not receive care at their place of choice, and instead experience repeated admissions to hospitals and emergency rooms [8, 9]. Aggressive treatment at the end of life is costly and has a negative impact on well-being and satisfaction of patients and families [10–15].

Palliative care seeks to improve the quality of life of patients and their families facing advanced progressive illness through pain and symptom management, communication and support for goals of care, and coordination of care as patients transition between care settings at the end of life [16]. Earlier access to palliative care in the cancer treatment trajectory has been shown to have beneficial outcomes to patients and their families [17–20]; yet most cancer patients in Singapore are referred to a palliative care provider only in the last few weeks of life or not at all [21, 22]. To address these shortcomings, a workgroup was commissioned by the Singapore Ministry of Health to develop a strategic plan for improving the end-of-life experiences of patients, including developing and enhancing palliative care services systematically across the country. The plan recommended identification of patients’ specific needs, as well as assessment of continuity of care and affordability of palliative care [22].

The research project *Cost of Medical Care of Patients with Advanced Serious Illness in Singapore* (COMPASS), funded by the Singapore Millennium Foundation and led by the Lien Centre for Palliative Care, aims to respond to this recommendation by establishing a cohort study to prospectively follow patients with advanced cancer until death, together with their informal primary caregivers. The primary objectives of the cohort study are to describe trajectories of quality of life of advanced cancer patients and their caregivers, and to quantify healthcare utilization and costs of patients as they transition between care settings. Secondary objectives are to investigate patient and caregiver awareness of and preferences for diagnostic and prognostic information, preferences in decision-making and end-of-life care, caregiver burden, perceived quality of care and to explore how these change as illness progresses, and finally to measure bereavement adjustment (Refer to Table 2 for the full list of research aims). To build a comprehensive understanding of the experience of patients and their families, the study will acquire cost and health utilization data as patient’s transition between care settings at the end of life. In order to promote transparency in detailed methods, analysis and reporting plans of COMPASS, we present here the full study protocol.

## Methods

### Study setting

This is a cohort study of 600 patients with advanced cancer, their primary informal caregivers (hereafter referred to as ‘caregivers’) and their treating medical oncologists conducted at two tertiary institutions providing oncology treatment in Singapore: National Cancer Centre and National University Hospital. The National Cancer Centre alone treats almost 70% of the public sector oncology cases [23]. Patients with advanced cancer are recruited from outpatient clinics at medical oncology departments of these two institutions and are surveyed every 3 months until death. Caregivers are also surveyed every 3 months and will additionally be assessed at 8 weeks and 6 month post-patient death. Follow up surveys may be conducted in settings other than outpatient clinics, as the study follows patients as they transition across different care settings.

### Participants

The study recruits Singapore citizens or Permanent Residents aged 21 years (age of majority) or older with a diagnosis of advanced solid cancer (stage IV). For patients with a diagnosis of breast or prostate cancer, we have the additional inclusion criteria of metastasis to an organ site, as breast and prostate patients typically have better prognoses that are likely to surpass the duration of the study [24, 25]. Patients with Eastern Cooperative Oncology Group (ECOG) performance status ≤2 are included to ensure adequate functional status for participation [26, 27]. Caregiver participants are required to be a primary informal caregiver of the patient, which is defined as 1) one of the main persons providing care to the patient (e.g. accompanying patient for doctor’s visits, helping the patient with day to day activities) or, 2) one of the main persons ensuring provision of care (e.g. supervision of those who provide care, such as foreign domestic workers, which is a common practice in Singapore for patients at the end of life) or, 3) main person or one of the main persons involved in making treatment decisions on behalf of the patient [28]. Caregivers who are foreign domestic workers are excluded. Patients’ medical oncologists are also recruited into the study.

### Recruitment and data collection

**Fig. 1:**
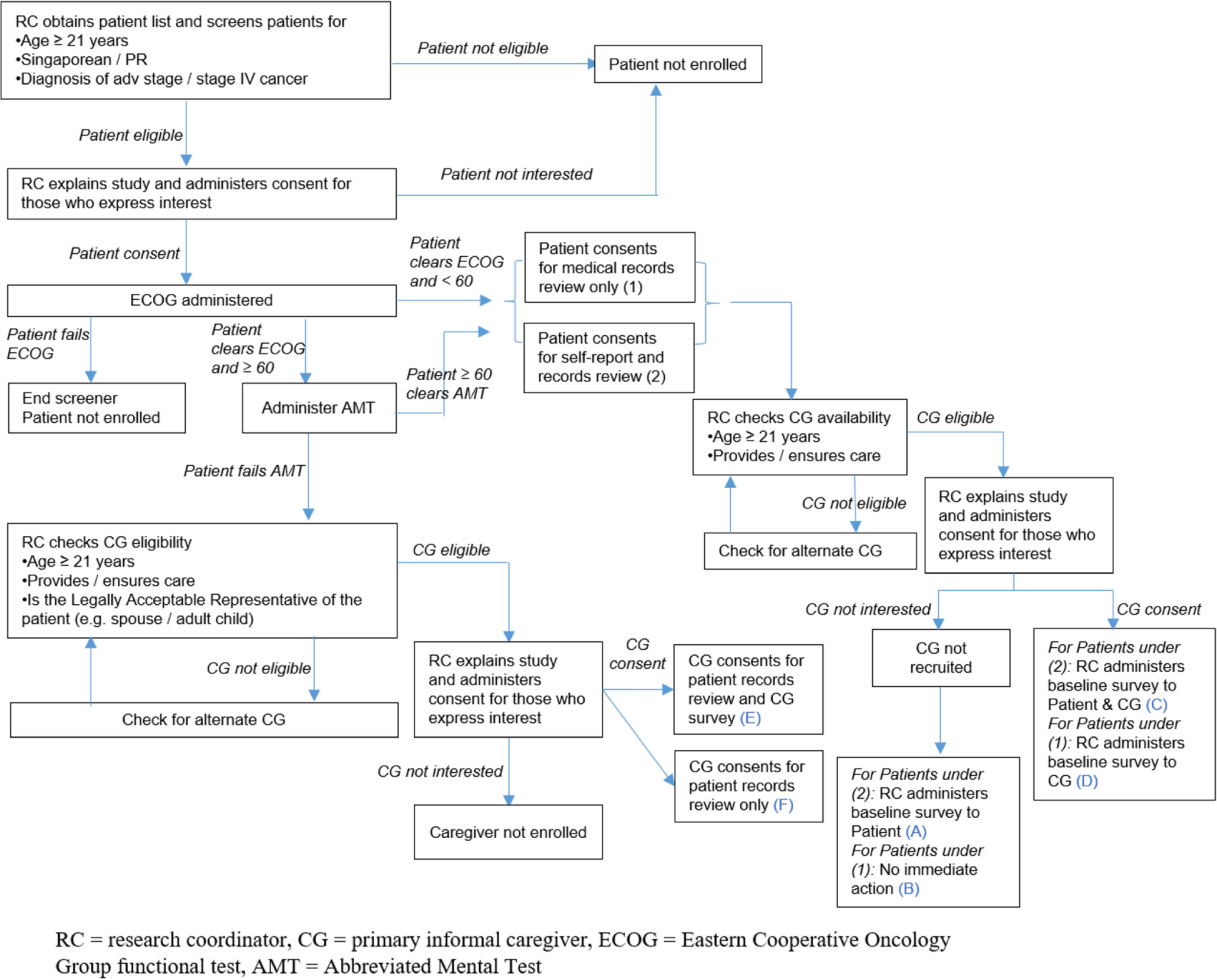
COMPASS recruitment flow and administration of baseline survey

The study protocol and all study-related documents were approved and are monitored by the SingHealth Centralised Institutional Review Board. The study is registered at www.clinicaltrials.gov (NCT02850640) on June 9, 2016 prior to study recruitment that commenced on July 8, 2016. Patients are pre-screened for eligibility (i.e., citizenship status, age, diagnostic criteria) and those who meet study inclusion criteria are approached by a research coordinator during their oncology visit for participation in the study. Patients are informed that their participation is voluntary and does not affect healthcare services received, that their identifying information will be kept confidential and that the study results will be reported in aggregate form. Patients who agree to participate will have their caregiver approached for recruitment. Both patients and their caregivers who agree to participate will provide written informed consent. For patients who are not able to give their consent due to cognitive status (as determined by medical records or through the Abbreviate Mental Test administered to participants ≥60 years), their caregiver is asked if they are willing to consent on behalf of the patient for medical/ billing records review of the patient. All patients who consent are administered the ECOG scale to determine their performance status (cut-off score to be included in study ≤2). Refer to Fig. 1 for the study recruitment process.

Consent forms, screeners and surveys for both patients and caregivers are administered in his/her preferred language out of the following options: English, Mandarin, and Malay. These three languages cover 99.5% of language literacy in Singapore [29]. Patients complete surveys that are interviewer-administered (i.e., interviewer reads the questions and answer options out to the patient), while caregivers have the option of doing it on their own; mode of survey administration is captured. Data from both patient and caregiver participants are captured electronically using an online survey platform. Participants are identified by a study code. There are several ways patients and caregivers can participate in the study, depending on their eligibility and interest. Categories A-F in Table 1 indicates the type of data that are obtained from the participant (and their caregiver) based on their category of participation.

**Table 1:** Types of data from the patient (and their caregiver) based on cognitive status and consent

Category	Cognitive status	Study Participant(s)	Type of data consented to
A	Patient is < 60 years of age or patient is ≥60 and passed the AMT	Patient (Caregiver declined /not available)	1. Patient self-report survey 2. Patient records review
B		Patient (Caregiver declined /not available)	1. Patient records review
C		Patient and caregiver	1. Patient self-report survey 2. Patient records review 3. Caregiver self-report survey
D		Patient and caregiver	1. Patient records review 2. Caregiver self-report survey and information as proxy for patient
E	Patient is ≥60 and failed the AMT	Patient (consent from legal representative) and caregiver	1. Patient records review 2. Caregiver self-report survey and information as proxy for patient
F		Patient (consent from legal representative)	1. Patient records review

*AMT* Abbreviated Mental Test, which is a cognitive screener with age- and education-determined cut off scores, *records review* review of medical and billing data

To minimize attrition rates we are conducting face-to-face follow up assessments at participants’ preferred locations (e.g., at outpatient clinic, at home, or at step-down care institution). Patients’ medical and billing records are acquired from all locations that provide care to enrolled patients (e.g., inpatient/outpatient hospital, pharmacy, hospice). Study participants will be compensated with a $25 voucher for completion of each survey. This study was initiated in July 2016.

### Outcome measures

Patients and caregivers will complete a survey packet assessing a broad range of topics regarding their quality of life, healthcare utilization/ expenditure, and perceived quality of care. Oncologists who participate in this study will complete a survey assessing their awareness, beliefs and practices regarding palliative services and end-of-life care. Medical and billing records are accessed for patient diagnostic, treatment information, and costs. Data from the physician survey and medical records are linked to information from the patient/caregiver surveys. The following outcome measures are administered in the study:

1. *Quality of life.* Quality of life in this study encompasses patient reported outcomes of physical symptoms and functioning as well as psychosocial well-being. The Functional Assessment of Cancer Therapy –General (FACT-G) [30] is a 27-item measure of quality of life of cancer patients. This is an extensively used instrument and comprises of four subscales: physical well-being, functional well-being, emotional well-being, and social well-being. Seven items drawn from the palliative symptom specific subscale [31] are administered to measure symptom burden. Equivalence between English and Chinese version for the FACT-G has been reported using a Singaporean sample [32]. A modified version of the Brief Pain Inventory (BPI) [33] is used to assess the severity of pain among patients and the degree to which their pain interferes with common dimensions of feeling and function. Activities of daily living (ADL) in patients are assessed using the Physical ADL section of the Older American Resources and Services (OARS) Activities of Daily Living questions [34].
2. *Healthcare utilization and expenditures.* Health care utilization from hospitals will be obtained through a combination of self-report and billing data, and will consist of total number of hospitalizations, visits to emergency department, chemotherapy and radiotherapy visits. Health care expenditures are classified by source of payment: inpatient, outpatient, pharmacy, emergency department, expenses on alternative medicines, and combined. Palliative care utilization (including those from transitions between settings) are also obtained through a combination of patient self-report and medical/ billing records. Patients will initially be asked where they received palliative care treatment, and billing records will then be traced at those institutions.
3. *Awareness of and preferences for information and for treatments.* Patient and caregiver awareness of and preferences for diagnostic and prognostic information are assessed by asking questions developed specifically for the study and adapted from Cancer Care Outcomes Research and Surveillance (CanCORS) Consortium and the Study to Understand Risks, Priority and Issues at End-of-Life (SURPRISE) [35]. Items include the extent participants understand the severity of the patient’s condition, whether patient’s treatment is perceived to cure patient’s condition or extend their lives, whether they would like to know how long patient is likely to live under various treatment options, and how long they think the patient is likely to live.
4. *Awareness and utilization of palliative care services*. Patients and caregivers will be asked about their awareness and their source of learning about palliative care services. The utilization of palliative care and the reasons for not using the services will be documented. These questions have been adapted from patient survey, version 7.0, used in observational study conducted by CanCORS Consortium [36]. Treating medical oncologists will be asked the timing of discussion regarding access to palliative care with patients.
5. *Preferences for treatments and decision-making.* Patients and caregivers are asked about their preference for pain relief /treatment cost versus life-extending treatments. They will also be asked their involvement in decision making and the extent to which patients’ preferences for involvement in decision making are met. The questions on decision making have been adapted from patient survey, version 7.0, CanCORS [36]. In addition, medical oncologists are asked about who makes the final treatment decision for their patients.
6. *Quality of care.* Patient and caregiver’s perceived quality of care (e.g. coordination of care, confidence in providers, treatment information, health information, access to cancer care, psychosocial care and symptom control) are assessed using a scale used by Ayanian et al. [37] that consists of 13 questions addressing specific problems of care and one question eliciting overall rating of care on a five-point scale ranging from poor to excellent. (Cronbach α’s = 0.81–0.86).
7. *Psychological distress.* Patient and caregiver psychological distress (defined as depression and anxiety) are assessed using the Hospital Anxiety and Depression Scale (HADS) [38]. The HADS has fourteen items that yield two subscales: anxiety and depression. This instrument has a Singapore Mandarin version that has been reported to be valid and reliable (Cronbach α’s = 0.74–0.85) to use in cancer patients in Singapore [39].
8. *Caregiver burden.* The modified Caregiver Reaction Assessment scale, which consists of 21 items are used to measure caregiver burden. The instrument yields four subscales: schedule and health, finances, family support and esteem. We will use the modified version that has been validated for use in Singapore and reported to be reliable (Cronbach α’s = 0.66–0.82) [40].
9. *Caregiver’s perception of the patient’s end of life.* Caregivers are assessed at 8-weeks post patient death on their and patient’s experience at the end of life using the 13-item Caregiver Evaluation of the Quality of End-Of-Life Care (CEQUEL). The CEQUEL is a valid and reliable (Cronbach α’s = 0.52–0.78) [41] measure of quality of end-of-life care from the caregiver’s perspective which includes measures of perceived suffering and prolongation of death.
10. *Caregiver bereavement adjustment.* Caregivers are assessed at 6-months post patient death for bereavement adjustment using the 5-item Brief Grief Questionnaire [42]. The reliability and validity of this instrument has been reported (Cronbach α = 0.75) [43].

All surveys that do not have Mandarin and Malay versions were translated with the assistance of a professional translation service. Cognitive interviews were conducted with ten participants in each language to check the readability of the translated questionnaire.

### Data analytic plan

#### Sample size calculation

The sample size calculation was not based on a single endpoint, but rather to enrol an appropriate number of subjects that ensures sufficient precision and power in answering the research questions while maintaining feasibility. A sample of 600 advanced cancer patients, subject to 20% loss to follow-up will allow 95% confidence intervals for proportions to be estimated with a margin of error of less than 0.05 and for means to be estimated with a margin of error of less than 10% of the associated observation standard deviation. Further, at α = 0.05, this sample size will provide greater than 80% power for comparison of proportions differing by at least 0.20 between groups and for comparison of means differing by at least 0.4 standard deviations between groups, under the assumption that the smaller group has at least 100 subjects.

## Discussion

The primary objective of this study is to describe trajectories of quality of life of advanced cancer patients and their caregivers, and to quantify healthcare utilization and costs of patients as they transition between care settings at the end of life. This is a cohort study for advanced cancer patients in Singapore that is distinctive as it follows patients as they transition across care settings and acquires data from various sources. It is a large undertaking, with coordinated effort of two tertiary hospitals providing oncology care, and data sharing from various sources of information including the hospices that patients transition to and the national registry of deaths. However we believe this study is important in presenting a comprehensive picture of the end-of-life experience of advanced cancer patients and their families. Table 2 summarizes the research aims and their policy and clinical significance.

**Table 2:** Research aims and significance of the COMPASS study

Research aims	Public health policy/clinical significance
1. To describe trajectories of patient and caregiver quality of life	❖ Identify modifiable risk factors ❖ Plan specific end-of-life care services to address deterioration in these quality of life domains
2. To quantify healthcare utilization and medical expenditures of patients at the end of life	❖ Identify predictors of high healthcare utilization and expenditure ❖ Plan for healthcare spending and subsidies
3. To describe patient reported pain and attitudes toward pain management at the end of life	❖ Inform understanding of patient experiences to optimize cancer pain management
4. To examine patient and caregiver awareness of and preferences for diagnostic and prognostic information	❖ Inform the extent of patient and caregiver awareness and the need to increase patient and caregiver understanding
5. To examine preferences for treatment and decision-making at the end of life	❖ Inform the extent patient preferences for treatments and decision-making are met and how practice can be improved
6. To examine awareness and utilization of hospice palliative care services	❖ Identify barriers to palliative care use and potential need for patient/ public education
7. To examine patient utilization of complementary and alternative therapies and their purpose	❖ Inform the extent, purpose, and costs ❖ Identify whether use of these therapies are barriers to seeking medical treatment and potential for patient education
8. To describe patient transitions between healthcare settings at the end of life	❖ Inform and improve on continuity of end-of-life care
9. To examine patient and caregiver perceived quality of care at the patient’s end of life	❖ Inform and improve quality of specific areas of end-of-life care
10. To describe caregiver bereavement adjustment	❖ Identify risk factors for caregiver bereavement adjustment difficulty ❖ Inform bereavement support service delivery

The data from this cohort study of advanced cancer patients and their caregivers will yield important information for improving the end-of-life experience of patients and their caregivers. Greater understanding of these trajectories can help us plan specific end-of-life care interventions or improve existing services. For instance, if we find that most patients deteriorate in certain quality of life domains (e.g. emotional well-being) several months before their death, then it implies that earlier intervention to address psychological distress may be useful. Studying the trajectories of patient quality of life will also allow identification of modifiable protective/risk factors that can be targeted through interventions. If characteristics of caregiver (e.g., level of caregiver competency, amount of time spent with patient) are found to predict patient’s physical and functional well-being, then interventions to enhance caregiving abilities may be undertaken to improve patient quality of life.

The data generated to meet our study objectives also carry direct public policy relevance. The perceived quality of care reports of patients and caregivers can be useful to identify areas of oncologic and end-of-life care that may be targets for improvement at the national level. The extent to which patients and caregivers are aware of the role and option of palliative care services can be important to consider in identifying strategies to increase awareness, both at patient and public levels. Mapping patients as they transition between care settings toward the end of life and their experiences during these transitions can also provide valuable information on improving continuity of care for patients seeking palliative care. Also, quantifying costs of healthcare utilization will allow for planning of governmental spending on palliative healthcare and planning subsidies, setting budgets and making recommendations for how much patients should save for end-of-life care, or for planning prospective payments and other incentive strategies aimed to reduce care transitions and improve treatments.

There are a few foreseeable challenges to consider in the proposed study. Attrition may be a problem due to patient’s expected declining functional status. We plan to follow patients as they transition through healthcare settings (e.g., hospital to hospice homecare to inpatient hospice) and will contact them for assessments every three months. To minimize attrition, we built flexibility in the data collection protocol by conducting assessments at locations convenient for patients. We also allow caregivers to answer questions regarding healthcare utilization on the patient’s behalf (e.g., number of hospital admissions in the last month) if the patient is too ill to participate. Another challenge is coordination of recruitment and data abstraction from electronic medical/ billing records collection across multiple sites given a lack of centralized system or database. Although being a relatively small country, Singapore has six regional health systems that is in the process of being re-organized into three integrated clusters. Both study sites are located in separate clusters, which means they each require a separate research collaboration agreement, are accountable to separate review boards, and have different patient systems. To address this challenge, PIs for each study site will provide guidance on navigation within their own healthcare system. The team of data collectors, however, are positioned centrally at the Lien Centre for Palliative Care for standardization and monitoring of data collection procedures.

In summary, the COMPASS study is a cohort study of advanced cancer patients and their caregivers that is expected to generate robust data to provide an understanding of patients’ experience and their healthcare at the end of life in Singapore. The study will elucidate the experiences, preferences of patients and caregivers that will be used to identify predictors of satisfactory and affordable care at the end of life for patient and their families. The data generated from this study will also be used to identify interventions and policies aimed at improving the end-of-life experience and palliative care services that are sustainable to healthcare providers funding healthcare for patients in Singapore and beyond.

## Abbreviations

ADLs: Activities of daily living
AMT: Abbreviated Mental Test
BPI: Brief Pain Inventory
CanCORS: Cancer Care Outcomes Research and Surveillance
CEQUEL: Caregiver Evaluation of the Quality of End-Of-Life Care
COMPASS: Cost of Medical Care of Patients with Advanced Serious Illness in Singapore
ECOG: Eastern Cooperative Oncology Group
FACT-G: Functional Assessment of Cancer Therapy –General
HADS: Hospital Anxiety and Depression Scale
OARS: Older American Resources and Services
SURPRISE: Study to Understand Risks, Priority and Issues at End-of-Life
